# Andrographolide Induces Noxa-Dependent Apoptosis by Transactivating ATF4 in Human Lung Adenocarcinoma Cells

**DOI:** 10.3389/fphar.2021.680589

**Published:** 2021-04-29

**Authors:** Junqian Zhang, Chunjie Li, Li Zhang, Yongqing Heng, Tong Xu, Yunjing Zhang, Xihui Chen, Robert M Hoffman, Lijun Jia

**Affiliations:** ^1^ Cancer Institute, Longhua Hospital, Shanghai University of Traditional Chinese Medicine, Shanghai, China; ^2^ Department of Surgery, University of California, San Diego, La Jolla, CA, United States; ^3^ Anticancer Inc., San Diego, CA, United States

**Keywords:** TCM, andrographolide, lung adenocarcinoma, apoptosis, ATF4, noxa

## Abstract

Lung adenocarcinoma is the most common pathological type of lung cancer with poor patient outcomes; therefore, developing novel therapeutic agents is critically needed. Andrographolide (AD), a major active component derived from the traditional Chinese medicine (TCM) *Andrographis paniculate*, is a potential antitumor drug, but the role of AD in lung adenocarcinoma remains poorly understood. In the present study, we demonstrated that AD inhibited the proliferation of broad-spectrum lung cancer cell lines in a dose-dependent manner. Meanwhile, we found that a high dose of AD induced Noxa-dependent apoptosis in human lung adenocarcinoma cells (A549 and H1299). Further studies revealed that Noxa was transcriptionally activated by activating transcription factor 4 (ATF4) in AD-induced apoptosis. Knockdown of ATF4 by small interfering RNA (siRNA) significantly diminished the transactivation of Noxa as well as the apoptotic population induced by AD. These results of the present study indicated that AD induced apoptosis of human lung adenocarcinoma cells by activating the ATF4/Noxa axis and supporting the development of AD as a promising candidate for the new era of chemotherapy.

## Introduction

Lung adenocarcinoma is recalcitrant cancer with overall survival of less than 5 years (Denisenko et al., 2018). Intolerable side effects and multidrug resistance are still the main causes of the poor outcomes of patients with lung adenocarcinoma (Malhotra and Perry, 2003; Thomas et al., 2015; Jamal-Hanjani et al., 2017; Rotow and Bivona, 2017). Therefore, it is urgently needed to develop novel therapeutic agents with high efficiency and low toxicity to ameliorate patient outcomes.

Cell apoptosis is a process of programmed cell death, and inducing tumor cell apoptosis has become a strategy for cancer therapy (Gerl and Vaux, 2005; Hanahan and Weinberg, 2011). Among the apoptotic regulatory proteins, pro-apoptotic BH3-only (Bcl-2 homology domain 3) protein Noxa, a member of the Bcl-2 (B cell lymphoma-2) family proteins, has been defined as an antitumor drug target (Pérez-Galán et al., 2006; Albert et al., 2014; Guikema et al., 2017; Morsi et al., 2018). Inoue et al. reported that Noxa mediated HDAC (histone deacetylase) inhibitor-induced apoptosis and suggested that activated Noxa could be a potential clinical target for chronic lymphocytic leukemia and lymphoma therapy (Inoue et al., 2007). Shibue et al. reported that Noxa is necessary for irradiation-induced apoptosis and supported that upregulated Noxa may provide a new strategy for cancer therapy (Shibue et al., 2003). Activating transcription factor 4 (ATF4) is a universal stress-responsive gene and could transcriptionally activate Noxa in response to chemotherapy. Armstrong et al. reported that apoptosis induced by fenretinide and bortezomib via upregulating Noxa was dependent on ATF4 (Armstrong et al., 2010). Chen et al. found the same mechanism in a small molecule compound MLN4924-treated human esophageal cancer cells (Chen et al., 2016). In conclusion, targeting ATF4/Noxa axis could be a promising strategy for cancer treatment (Zhu et al., 2012; Nunez-Vazquez et al., 2020).

There is currently a great interest in developing traditional Chinese medicine (TCM) into first-line therapy for cancer (Tang et al., 1999; Efferth et al., 2007; Lu et al., 2019). Andrographolide (AD) is one of the major active components of the TCM *Andrographis paniculate* (Zhao et al., 2002). Previous studies have reported that AD exhibits a broad-spectrum antitumor efficacy in various cancer cells (Banerjee et al., 2016; Kumar et al., 2016; Islam et al., 2018; Chen et al., 2019). However, the antitumor efficacy and the underlying molecular mechanisms of AD on human lung adenocarcinoma cells remains poorly understood. In this study, we demonstrated that AD exhibits a broad-spectrum proliferation inhibitory effect in lung cancer cells, and firstly reported that AD induced lung adenocarcinoma cell apoptosis via activating ATF4/Noxa axis. The present study provides a basis to clinically develop AD for the new era of chemotherapy.

## Materials and Methods

### Cells and Reagents

Human lung adenocarcinoma cells (A549, H1299), human lung squamous cells (SK-MES-1), murine Lewis lung carcinoma cells (LLC), and normal human bronchial epithelial cells (BEAS-2B) were obtained from the American Type Culture Collection (Manassas, VA, United States). These cells were cultured in Dulbecco’s modified Eagle’s medium (DMEM, BasalMedia, Shanghai, China) which contains 10% fetal bovine serum (FBS, BasalMedia, Shanghai, China) and 1% penicillin–streptomycin solution (BasalMedia, Shanghai, China) at 37°C with 5% CO_2_. Andrographolide (Sigma-Aldrich, Germany) was dissolved into a concentration of 100 mM with dimethyl sulfoxide (DMSO, Sigma-Aldrich, Germany) and stored at -20°C. Antibody against β-actin was obtained from HuaBio (Hangzhou, China); Antibodies against cleaved-PARP (c-PARP), PARP, cleaved-caspase-3 (c-Casp3), caspase3 (Casp3), ATF4, c-Myc, Noxa, Puma, Bid, Bim, Bik, Bax, and Bak were all obtained from Cell Signaling Technology (Beverly, MA, United States).

### Cell Proliferation Assay

Cells in the exponential growth phase were seeded in ATPlite plates in triplicate, 2000 cells per well, cultured overnight, and treated with 1‰ DMSO or AD at indicated concentrations for 24 h and 48 h, followed by the ATPlite luminescence assay (BD Pharmingen, Franklin Lakes, New Jersey, United States).

### Western Blotting

Cells were harvested and lysed in RIPA buffer (Beyotime, Shanghai, China). Protein concentrations were determined by the protein assay kit (Epizyme, Shanghai, China). Gel electrophoresis (SDS–PAGE) was used to separate the total proteins of the samples. Then, the proteins were transferred onto a polyvinylidene fluoride membrane (PVDF). 5% nonfat milk in TBST was used to block the PVDF membrane for 1 h at room temperature. The membranes with a primary antibody were co-incubated at 4°C and washed three times with TBST overnight, and then co-incubated with a secondary antibody at room temperature for 1 h. After then, washed three times with TBST and visualized with the ECL kit (Share Bio, Shanghai, China) and film (Tanon, Shanghai, China).

### Real-Time Polymerase Chain Reaction Analyses (RT-PCR)

Ultrapure RNA kit (ComWin Biotech, Beijing, China) was used to isolate total RNA, and 1 μg total RNA was reversed to cDNA by using the PrimerScript reverse transcription reagent kit (Vazyme Biotech, Nanjing, China). Then, the cDNA was quantified with RT-PCR by using the Power SYBR Green PCR MasterMix (Vazyme Biotech, Nanjing, China) on the ABI 7500 thermocycler (Applied Biosystems, Foster City, CA, United States). The mRNA abundance of each sample was normalized to β-actin. Primers were designed and synthesized by BioSune (Shanghai, China). The sequences of the primers were as follows: human β-actin: forward 5′-CGT​GCG​TGA​CAT​TAA​GGA​GAA​G-3′ and reverse 5′-AAG​GAA​GGC​TGG​AAG​AGT​GC-3′; human ATF4: forward 5′-ATG​ACC​GAA​ATG​AGC​TTC​CTG-3′ and reverse 5′-GCT​GGA​GAA​CCC​ATG​AGG​T-3′; human Noxa: forward 5′-ACC​AAG​CCG​GAT​TTG​CGA​TT-3′ and reverse 5′-ACT​TGC​ACT​TGT​TCC​TCG​TGG-3′; and human c-Myc: forward 5′-GGC​TCC​TGG​CAA​AAG​GTC​A-3′ and reverse 5′-CTG​CGT​AGT​TGT​GCT​GAT​GT-3′.

### Apoptosis Assay

Cells were harvested after treatment with different concentrations of AD for 24 h and then stained by using Annexin V-FITC/PI stain kit following the manufacturer’s instructions (BD Pharmingen, New Jersey, United States). The stained cells were analyzed with flow cytometry (BD, New Jersey, United States).

### Gene Silencing Using Small Interfering RNA

The siRNA oligonucleotides were transfected into cells by using Lipofectamine 2000. Briefly, Opti-MEM (Invitrogen) was used to incubate with siRNA and Lipofectamine 2000 separately for 5 min at room temperature and mixed for 20 min, and then the mixture together with the serum-free medium were applied to the cells (final concentration of siRNA is 20 nM). All siRNAs were synthesized by GenePharma (Shanghai, China). The sequences of the siRNA were as follows: negative control: 5′-UUC​UCC​GAA​CGU​GUC​ACG​UTT-3′; siATF4: 5′-GCC​UAG​GUC​UCU​UAG​AUG​A-3′; siNoxa#1: 5′-GUA​AUU​AUU​GAC​ACA​UUU​C-3′ (Alves et al., 2006); and siNoxa#2: 5′-GGU​GCA​CGU​UUC​AUC​AAU​UUG-3′ (Yao et al., 2014).

### Statistical Analysis

Data were displayed as mean ± standard deviation. All data represented three independent experiments. Significant differences between groups were assessed by the two-tailed unpaired Student’s t-test of GraphPad Prism software. Four levels of significance were used for all tests (*p ≤ 0.05, **p ≤ 0.01, ***p≤ 0.001, and ***p≤ 0.0001).

## Results

### Andrographolide Inhibited the Proliferation of Lung Cancer Cells

**FIGURE 1:**
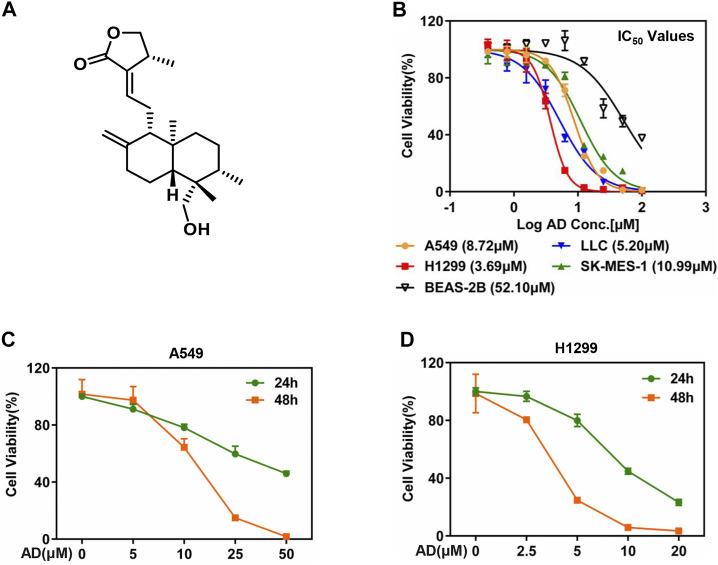
Andrographolide inhibited the proliferation of lung cancer cells. **(A)** The chemical structure of AD. **(B)** Cells were seeded in ATPlite plates in triplicate, 2000 cells per well, cultured overnight, and treated with 1‰ DMSO or various concentrations of AD (100, 50, 25, 12.5, 6.25, 3.13, 1.56, and 0.78 μM) for 48 h. The ATPlite cell viability assay was used to determine the half-maximal inhibitory concentrations IC_50_ of A549, H1299, SK-MES-1, LLC, and BEAS-2B cells, respectively. **(C–D)** Cells were seeded in ATPlite plates in triplicate, 2000 cells per well, cultured overnight, and treated with 1‰ DMSO or indicated concentrations of AD for 24 h and 48 h, followed by the ATPlite cell viability assay.

The chemical structure of AD is shown in Figure 1A. To evaluate the inhibitory efficacy of AD on the proliferation of lung cancer cells, we determined the half-maximal inhibitory concentration IC_50_ of AD in five cell lines including squamous carcinoma cells (SK-MES-1), adenocarcinoma cells (A549, H1299), murine Lewis lung cancer cells (LLC), and normal human bronchial epithelial cells (BEAS-2B) at 48 h. As shown in Figure 1B, the IC_50_ values were approximately 8.72, 3.69, 10.99, 5.2, and 52.10 μM in A549, H1299, SK-MES-1, LLC, and BEAS-2B cells, respectively. The results indicated that AD has a broad-spectrum inhibitory effect on lung cancer cells but has a weaker inhibitory effect on the normal human bronchial epithelial cells.

Considering the morbidity and prognosis of lung adenocarcinoma, we next focused on lung adenocarcinoma cells to explore the antitumor property of AD. First, we selected four concentrations of AD for further studies according to the IC_50_ values. Then, we examined the inhibitory effect of these concentrations of AD on the proliferation of the two lung adenocarcinoma cells. As shown in Figures 1C,D, AD significantly suppressed the cell proliferation of the two lung adenocarcinoma cells in a dose- and time-dependent manner.

### Andrographolide Triggered Apoptosis of Human Lung Adenocarcinoma Cells

**FIGURE 2:**
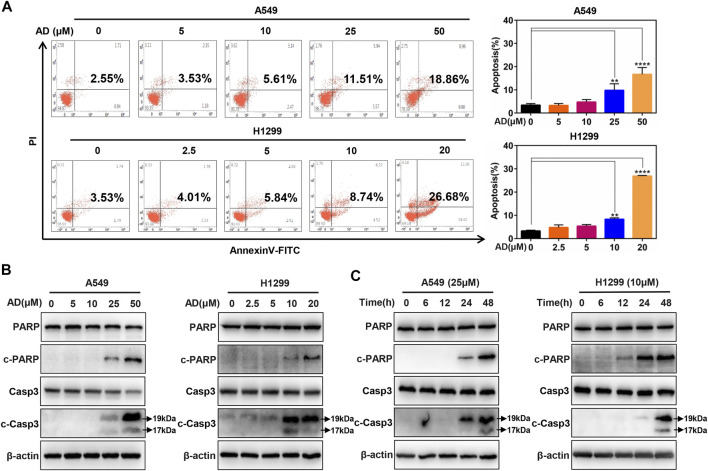
Andrographolide triggered apoptosis in human lung adenocarcinoma cells **(A)** Cells were treated with 1‰ DMSO or indicated concentrations of AD for 24 h, then subjected to Annexin V-FITC/PI staining, followed by the flow cytometric analysis. **(B–C)** After being treated with 1‰ DMSO or AD at indicated concentrations and different times, cell protein was extracted and detected by Western blotting with antibodies against PARP, c-PARP, Casp3 and c-Casp3, and β-actin (** *p* ≤ 0.01; **** *p* ≤ 0.0001).

To further investigate the underlying mechanisms of AD-induced cell growth inhibition, we determined cell apoptosis by Annexin V-FITC/PI stain and FACS in both A549 and H1299 cells after AD treatment for 24 h. As shown in Figure 2A, a significant dose-dependent increased apoptosis was detected in the two cells, especially in high concentration groups. Then, we examined c-PARP and c-Casp3 by immunoblotting to further confirm the induction of apoptosis by AD in A549 and H1299 cells. The results indicated that the expression levels of c-Casp3 and c-PARP were greatly increased in a dose- and time-dependent manner (Figures 2B,C). These findings demonstrated that AD induced apoptosis of the human lung adenocarcinoma cells.

### The BH3-Only Protein Noxa Was Upregulated by Andrographolide Treatment

**FIGURE 3:**
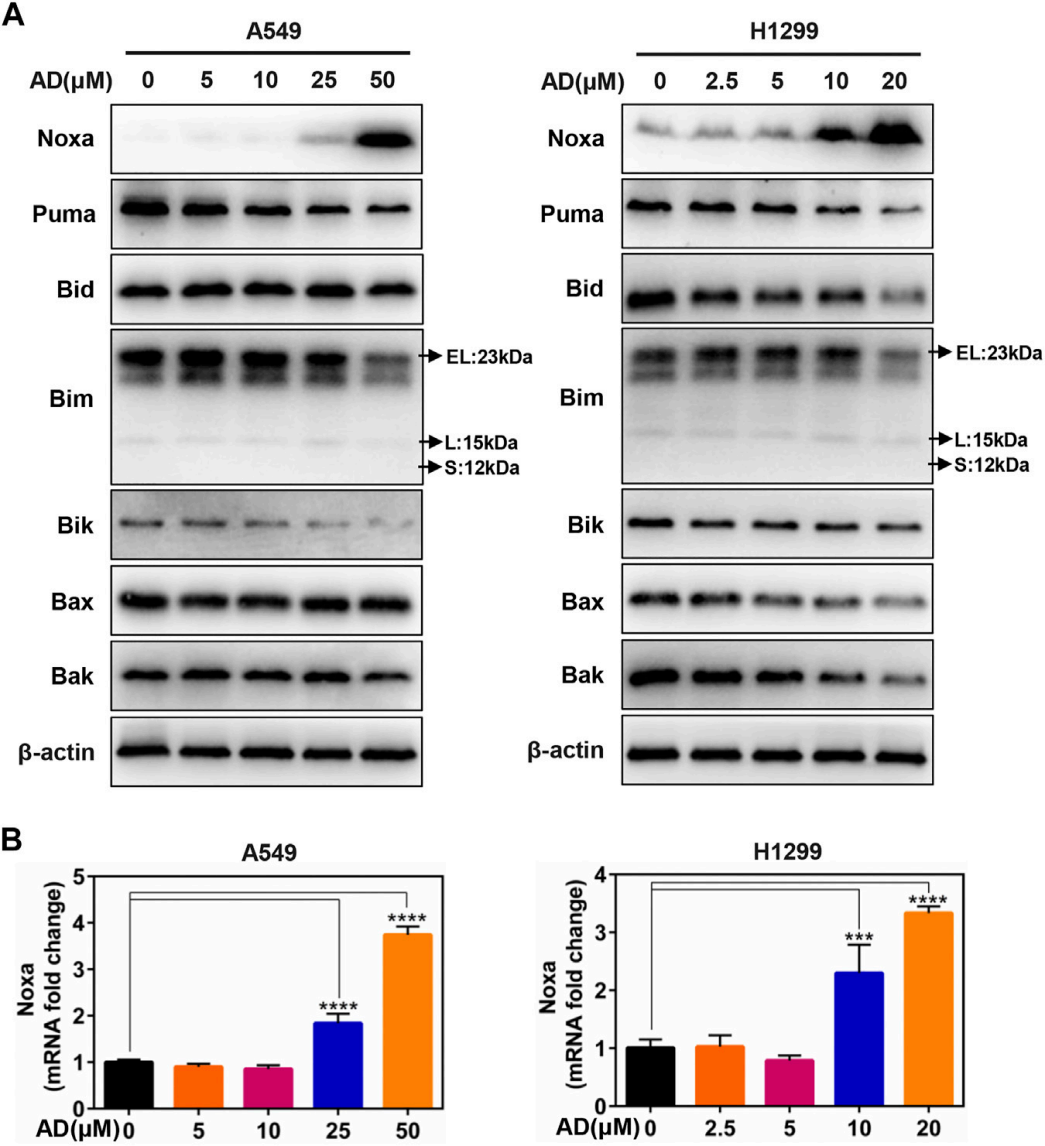
The BH3-only protein Noxa mediated andrographolide-induced apoptosis. **(A)** Cells were treated with 1‰ DMSO or the indicated concentrations of AD for 24 h. Cell protein was extracted and detected by Western blotting with antibodies against Noxa, Puma, Bid, Bim, Bik, Bax, Bak, and β-actin. **(B)** The mRNA level of Noxa was quantified by RT-PCR (normalized to β-actin) (*** *p* ≤ 0.001; **** *p* ≤ 0.0001).

Given that the role of the classical pro-apoptotic Bcl-2 family proteins in regulating apoptotic cell death, we next determined the expression of the pro-apoptotic proteins, including BH3-only proteins Puma, Bim, Bik, Bid, and Noxa, as well as their downstream pro-apoptotic proteins Bak and Bax in A549 and H1299 cells. These results indicated that only Noxa was significantly upregulated in the protein level in a dose-dependent manner (Figure 3A). Moreover, the mRNA level of Noxa was also substantially elevated by treatment of AD (Figure 3B). These findings indicated that Noxa was transcriptionally activated by AD treatment and suggested that Noxa plays a crucial role in AD-induced apoptosis.

### Noxa Knockdown Inhibited Andrographolide-Induced Apoptosis

**FIGURE 4:**
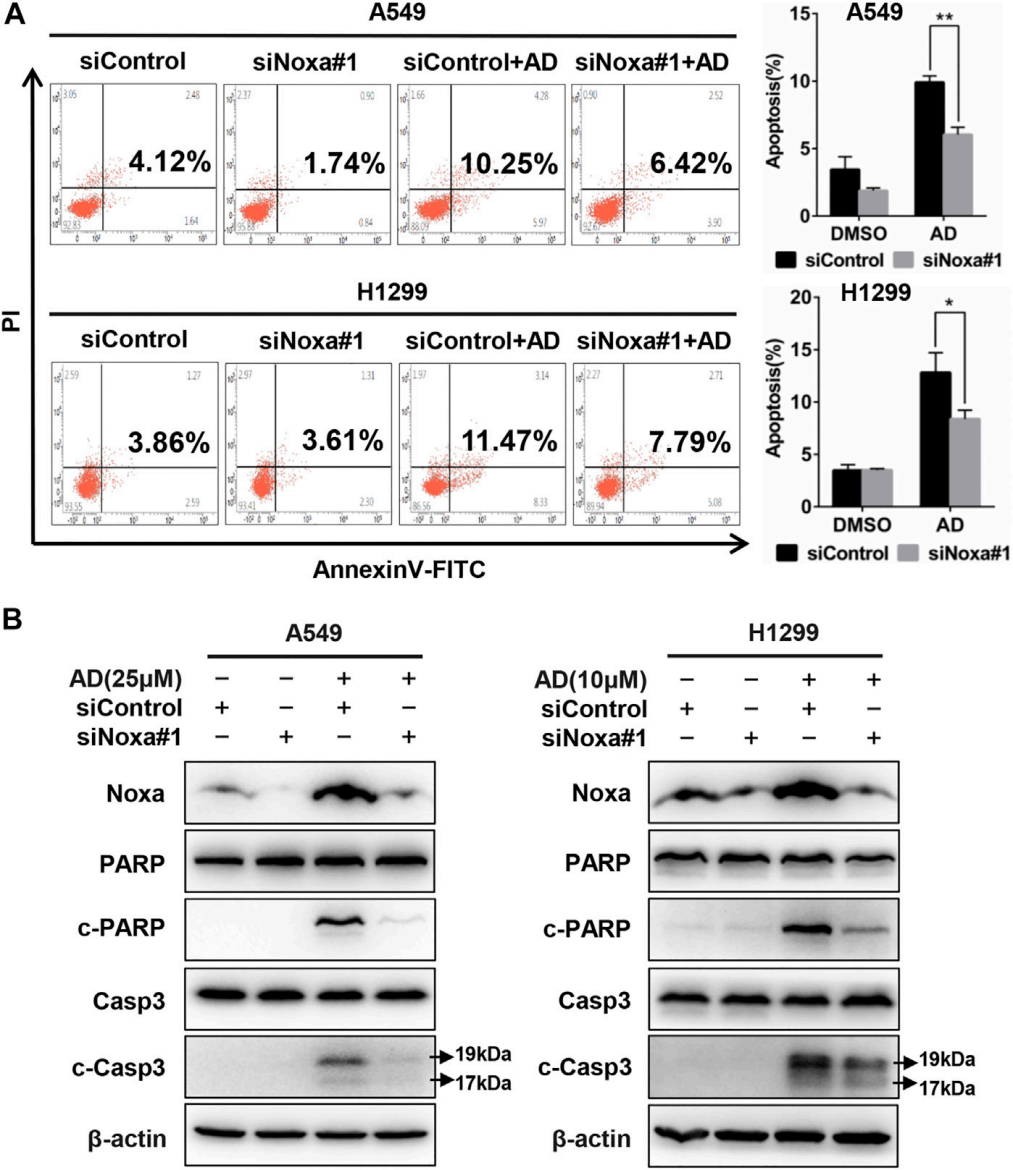
Noxa knockdown significantly decreased andrographolide-induced apoptosis in human lung adenocarcinoma cells. **(A)** Cells were transfected with siControl or siNoxa#1 and treated with 1‰ DMSO and AD (A549 25 μM and H1299 10 μM) for 24 h. Apoptosis was determined and quantified with Annexin V-FITC/PI staining analysis. **(B)** Cell protein was extracted and detected by western blotting with antibodies against Noxa, PARP, c-PARP, Casp3, c-Casp3, and β-actin (* *p* ≤ 0.05; ** *p* ≤ 0.01).

To confirm the role of Noxa in AD-induced apoptosis in A549 and H1299 cells, Noxa was knocked down by small interfering RNA (siRNA). As shown in Figure 4A and supplementary Figure S1A, Noxa knockdown significantly diminished AD-induced apoptosis as well as the expression levels of c-Casp3 and c-PARP (Figure 4B and Supplementary Figure S1B). These findings demonstrated that AD induced Noxa-dependent apoptosis of human lung adenocarcinoma cells.

### Andrographolide-Induced Apoptosis in Human Lung Adenocarcinoma Cells via ATF4/Noxa Axis

**FIGURE 5:**
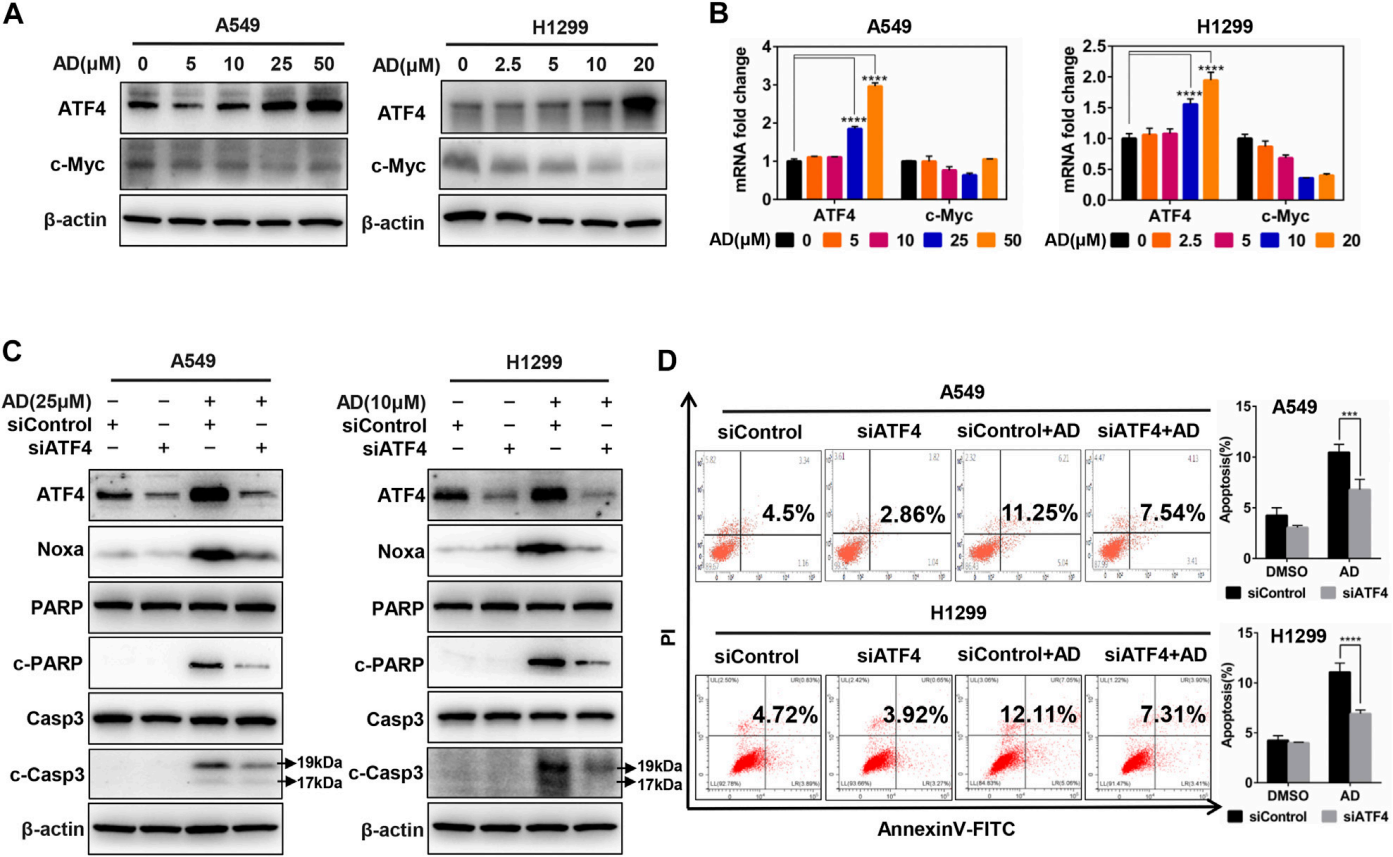
Andrographolide induced apoptosis in human lung adenocarcinoma cells via ATF4/Noxa axis. **(A–B)** Cells were treated with 1‰ DMSO or indicated concentrations of AD for 24 h; cell protein was extracted and detected by western blotting with antibodies against ATF4 and c-Myc. The mRNA level of ATF4 and c-Myc was quantified with RT-PCR (normalized to β-actin). **(C–D)** Cells were transfected with siControl or siATF4, then, treated with 1‰ DMSO or AD (A549 25 μM, H1299 10 μM) for 24 h, cell protein was extracted and detected by western blotting with antibodies against ATF4, Noxa, PARP, c-PARP, Casp3, c-Casp3, and β-actin. Apoptosis was determined and quantified by AnnexinV-FITC/PI staining analysis (* *p* ≤ 0.05; *** *p* ≤ 0.001; **** *p* ≤ 0.0001.).

Previous studies in our laboratory unveiled that ATF4 transcriptionally activated Noxa in esophageal squamous cell carcinoma cells after treated by small-molecule compound MLN4924 (Chen et al., 2016). In contrast, it was c-Myc but not ATF4 that transactivated Noxa to trigger cell apoptosis in head and neck squamous cell carcinoma cells after treated by MLN4924 (Zhang et al., 2019). To confirm the transcription factors responsible for Noxa induction upon AD treatment in both A549 and H1299 cells, we examined the protein and mRNA expression of c-Myc and ATF4. The results indicated that AD treatment strongly induced the upregulation of ATF4, but not c-Myc at both protein and mRNA levels (Figures 5A,B). Furthermore, ATF4 knockdown suppressed the transactivation of Noxa as well as the cleavage of PARP and caspase 3 in AD-treated A549 and H1299 cells (Figure 5C). Consistently, the apoptotic population of AD-treated cells was significantly diminished in both two ATF4 knockdown cells (Figure 5D). Taken together, AD transcriptionally activated ATF4, which induced the transcriptional activation of the pro-apoptotic protein Noxa to trigger apoptosis in both A549 and H1299 cells.

## Discussion

**FIGURE 6:**
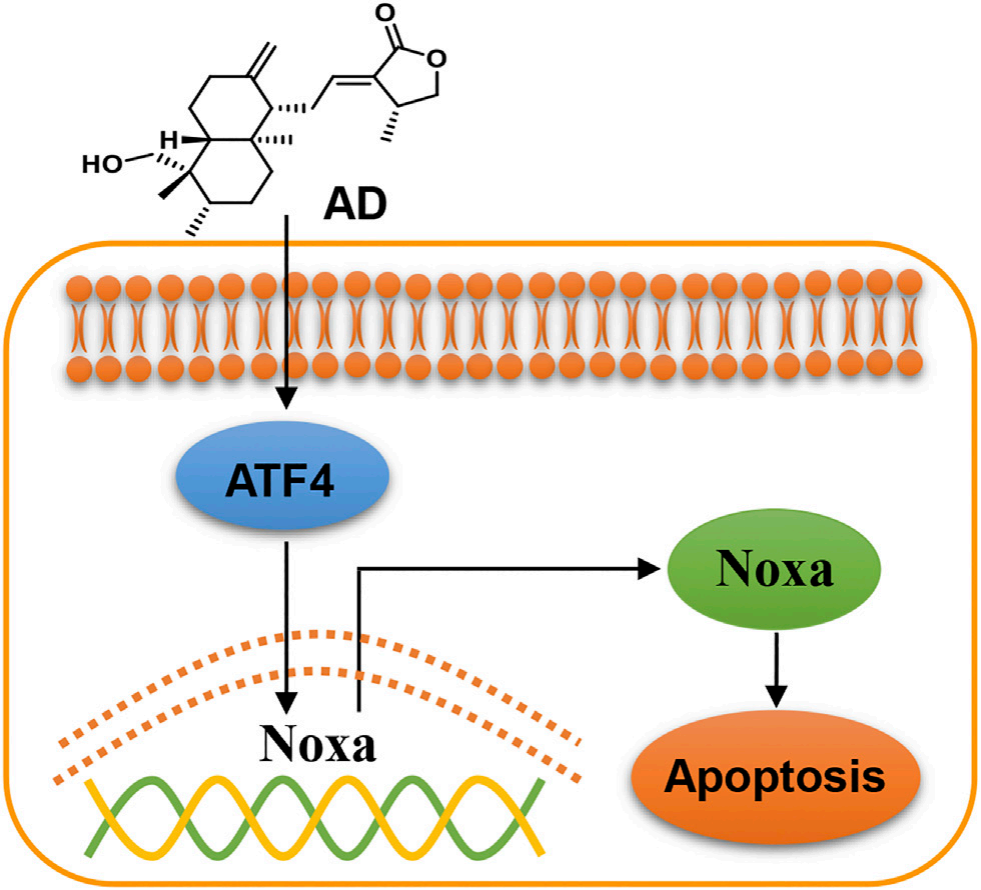
Working model of andrographolide-induced ATF4/Noxa-dependent apoptosis. AD triggered human lung adenocarcinoma cells apoptosis by transcriptionally activating ATF4 and subsequently transactivating the pro-apoptotic BH3-only protein Noxa.

In the present study, we found that AD exhibits a broad-spectrum inhibition of proliferation in lung cancer cells, and firstly demonstrated that AD activated the ATF4/Noxa axis to induce apoptosis of human lung adenocarcinoma cells (Figure 6). In recent years, emerging evidence has indicated that AD could exert an antitumor effect by inducing apoptosis of diverse cancer cells. Yang et al. reported that AD induced apoptosis of T-cell acute lymphoblastic leukemia cell (Jurkat) via inhibition of the PI3K/Akt pathway (Yang et al., 2016). Wu et al. reported that AD activated the LKB1/AMPK signal pathways and induced apoptosis of human nasopharyngeal carcinoma cells (C666–1) (Wu et al., 2018). Peng et al. also reported that AD induced apoptosis of nasopharyngeal carcinoma cells (HK1 and CNE-1) via inhibiting the NF-κB signal pathway (Peng et al., 2015). These findings collectively indicated that the mechanisms of AD-induced apoptosis may be cancer-type dependent.

While addressing the mechanisms of apoptosis induced by AD in human lung adenocarcinoma cells, we defined that the BH3-only protein Noxa mediated AD-induced apoptosis of human lung adenocarcinoma cells (A549 and H1299). BH3-only proteins Puma, Bim, Bik, Bid, Noxa, and their downstream proteins Bak and Bax all play critical roles in pro-apoptotic cell death (Gross et al., 1999; Cory et al., 2003; Breckenridge and Xue, 2004). It has been reported that some of the BH3-only proteins could mediate AD-induced cell apoptosis. Zhou et al. reported that AD promoted the cleavage of the BH3-only protein Bid to induce apoptosis of breast cancer cells (MDA-MB-231), cervical cancer cells (HeLa), and hepatoma cells (HepG2) (Zhou et al., 2006). Yang et al. reported that Bax and Bak are necessary for AD-induced apoptosis of lymphoma cells (Yang et al., 2010). In our study, we found that Noxa was significantly upregulated by AD in human lung adenocarcinoma cells, and Noxa knockdown significantly decreased AD-induced apoptosis. These results demonstrated that AD induced Noxa-dependent apoptosis of human lung adenocarcinoma cells.

The present study demonstrated that ATF4 transcriptionally activated Noxa in AD-treated lung adenocarcinoma cells. As a universal stress-responsive gene, ATF4 can be activated by several stimulations, such as oxidative stress, endoplasmic reticulum (ER) stress, and oxygen deprivation (Harding et al., 2003; Blais et al., 2004). A large number of studies indicated that activated ATF4 could induce cancer cell apoptosis for the oncotherapy (Jiang et al., 2013; Nunez-Vazquez et al., 2020; Xu et al., 2020). Zong et al. reported that radiation could upregulate ATF4 expression to induce cell apoptosis, and the overexpression of ATF4 could increase cell sensitivity to apoptosis in response to radiation (Zong et al., 2017). Sharma et al. found that cisplatin induced Noxa-dependent apoptosis through the upregulation of ATF4 in head and neck squamous cell carcinoma cells (Sharma et al., 2018). In this study, we found that both protein and mRNA levels of Noxa were upregulated after AD treatment, which suggested that Noxa was transcriptionally activated. Further studies showed that both the protein and mRNA levels of ATF4 were upregulated after AD treatment. Moreover, ATF4 knockdown inhibited the upregulation of Noxa as well as AD-induced apoptosis in human lung adenocarcinoma cells. Therefore, we confirmed that ATF4 transcriptionally activated Noxa in AD-induced apoptosis of human lung adenocarcinoma cells.

In summary, the present study firstly demonstrated that AD induced Noxa-dependent apoptosis by transactivating ATF4 in human lung cancer cells. These findings provided a scientific basis for developing AD as a promising candidate for the new era of chemotherapy.

## Data Availability

The original contributions presented in the study are included in the article/Supplementary Material, and further inquiries can be directed to the corresponding author.
