# Supplementary material for: Andrographolide Induces Noxa-Dependent Apoptosis by Transactivating ATF4 in Human Lung Adenocarcinoma Cells
Source: Front Pharmacol. 2021 Apr 29;12:680589. doi: 10.3389/fphar.2021.680589 (PMC8117100; doi:10.3389/fphar.2021.680589)
Supplement: Supplementary file 1 [file Image1.pdf]

Supplementary Material

1 Supplementary Figure

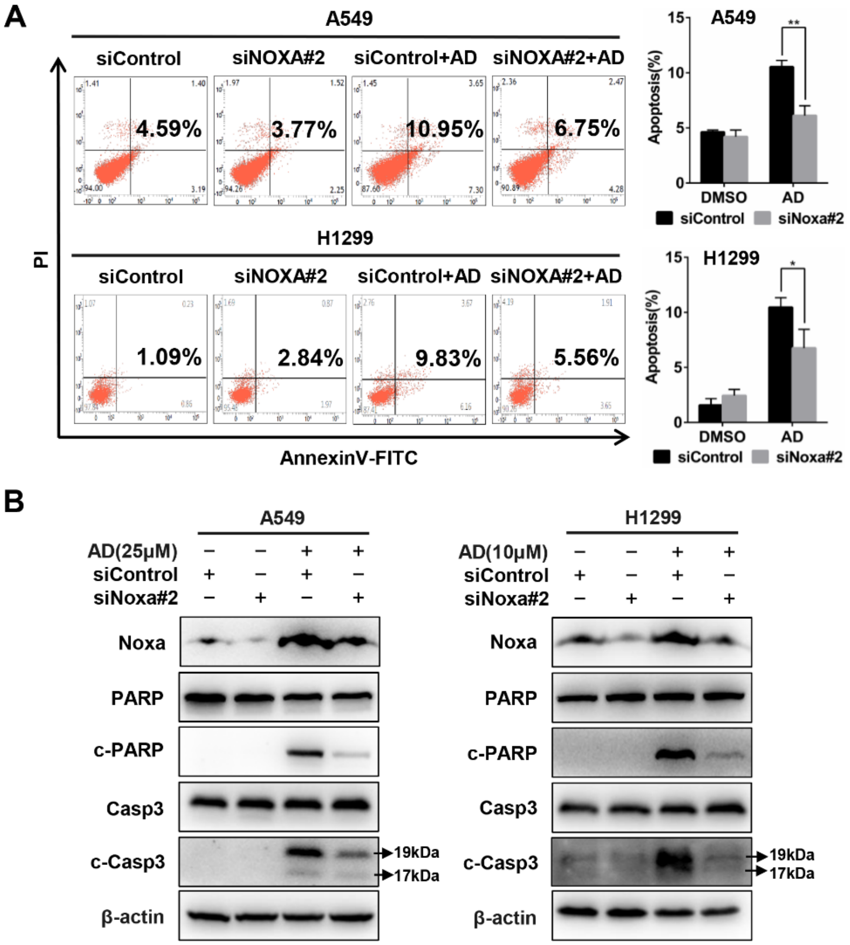

Supplementary Figure 1. Noxa knockdown significantly decreased Andrographolide-induced apoptosis in human lung adenocarcinoma cells. (A) Cells were transfected with siControl or siNoxa#2, and treated with 1‰ DMSO or AD (A549 25 μM, H1299 10 μM) for 24h. Apoptosis was determined and quantified with AnnexinV-FITC/PI staining analysis. (B) Cell protein was extracted and detected by western blotting with antibodies against Noxa, PARP, c-PARP, Casp3, c-Casp3 and β-actin. (*, $P \leqslant 0.05$; **, $P \leqslant 0.01$.).
